# Single-step rapid chromatographic purification and characterization of clinical stage oncolytic VSV-GP

**DOI:** 10.3389/fbioe.2022.992069

**Published:** 2022-10-28

**Authors:** Saurabh Gautam, Dongyue Xin, Alan Pardo Garcia, Bart Spiesschaert

**Affiliations:** ^1^ Boehringer Ingelheim International GmbH, Ingelheim, Germany; ^2^ ViraTherapeutics GmbH, Rum, Austria; ^3^ Boehringer Ingelheim Pharmaceutical, Inc., Ridgefield, CT, United States

**Keywords:** oncolytic viruses, cation-exchange chromatography, membrane adsorbers, sartobind S, downstream processing, VSV-GP, cryo-EM of viruses, virus analytics and characterization

## Abstract

Purification of viruses, especially for therapeutic purposes, is a tedious and challenging task. The challenges arise due to the size and surface complexity of the virus particles. VSV-GP is a promising oncolytic virus, which has been approved for phase I clinical trials by the Food and Drug Administration (FDA) of United States and Paul Ehrlich Institute (PEI) of Germany. The virus particles of VSV-GP are larger in size than vectors commonly used for gene therapy (e.g., adenovirus, adeno-associated virus, etc.). The current established proprietary clinical-grade manufacturing process for the purification of VSV-GP encompasses several chromatographic and non-chromatographic steps. In this study, we describe a new single-step purification process for the purification of VSV-GP virus, using cation exchange convective flow column with relatively higher yields. The purified virus was characterized for its quality attributes using TCID_50_ assay (for viral infectivity), host cell protein contaminant ELISA, SDS-PAGE, size exclusion chromatography (SEC), and cryo-electron microscopy. Furthermore, the purified viral therapeutic material was tested *in vivo* for its efficacy and safety. All these characterization methods demonstrated a therapeutic virus preparation of high purity and yield, which can be readily used for various studies.

## Introduction

Advanced therapeutic and medicinal products (ATMPs), often touted as the “golden bullet,” are a class of new and innovative therapeutics developed as breakthrough therapies for the treatment of “un-treatable or extraordinary diseases” such as against various types of cancer (Schneider et al., 2010). Oncolytic viruses are a class of ATMPs used to treat cancer by killing malignant cells, while sparing normal cells. Further discussion about oncolytic viruses can be read in these excellent publications (Muik et al., 2012; Kaufman et al., 2015; Macedo et al., 2020; Apolonio et al., 2021). Currently, there are only a limited number of oncolytic viral (OV) therapies being registered for commercial use in various international markets, of which IMLYGIC™ or Talimogene Laherparepvec (T-VEC) was the first OV to be approved both by FDA and EMA (EMA, 2018; Burke et al., 2021).

Vesicular stomatitis virus (VSV) is a negative-strand RNA virus that belongs to the family of rhabdoviridae (Barber, 2005). It can induce potent tumor cytotoxic effects, in a number of preclinical tumor models (Barber, 2004). VSV is also an important viral vector that has already been approved for human usage as the vaccine against the deadly Ebola viral infection (Hoenen et al., 2012; Sridhar, 2015). In this case, VSV was pseudotyped with Ebola surface glycoprotein thereby successfully demonstrating the principle of pseudotyping VSV with glycoprotein from a different virus.

Recently, a recombinant version (VSV-GP) has been developed as an immune-oncology platform that can induce both direct tumor cell killing as well as therapeutic cancer vaccination (Muik et al., 2012; Muik et al., 2014; Tober et al., 2014; Kimpel et al., 2018; Schreiber et al., 2019). Herein, VSV was pseudotyped with glycoprotein of lymphocytic choriomeningitis virus (LCMV), the pseudotyped virus being called as VSV-GP (Muik et al., 2012). This modification reduces its ability to induce anti-surface antibodies resulting in reduced sensitivity to complement-mediated neutralization and mitigates the inherent neurotropism of wildtype VSV (Muik et al., 2014; Kimpel et al., 2018). However, it must be noted that the obstacles arise both in early research as well as manufacturing of such innovative and revolutionary therapies.

One of the major challenges when developing a complex therapy, such as oncolytic viral therapy, lies in developing an efficient manufacturing process (Working et al., 2005; Ungerechts et al., 2016). This challenge is amplified by the need for a high drug concentration to allow for therapeutic dosing of oncolytic viruses as compared to vaccines (Heo et al., 2013; Ungerechts et al., 2016), wherein the dose requirement is several magnitude lower. Therefore, simple downstream processes involving 1–2 steps, used for viral vaccines (Kis et al., 2019), are generally not sufficient to achieve the quality and concentration. Consequently, an extensive downstream process is generally required to further concentrate the oncolytic virus and at the same time reducing the impurity (e.g., host cell protein and DNA) levels below the regulatory guidelines. This is a fine balance and generally difficult to achieve with simplified downstream processes used for vaccine applications.

There are various types of chromatography columns commercially available for downstream processing and purification of various therapeutic molecules, especially with ion exchangers (Jungbauer et al., 2009). However, it is important to understand that large virus-like particles or viruses have specific requirements when it comes to their purification (Cramer and Holstein, 2011; Bandeira et al., 2012; Kramberger et al., 2015; Singh and Heldt, 2022). Moreover, the largest challenges arise not only due to their size, but also due to the shape and complexity of surface charges on the viruses (Singh and Heldt, 2022). Furthermore, the surface charges on the virus particles are further modulated by incorporation of host cell proteins on the surface of virions (Burnie, 2022).

The challenges due to such complexities include but not limited to, the fact that virus size can be greater than the pore size of circular chromatography beads in the column (Singh and Heldt, 2022). Also, the shear stress generated, during the movement through the void volume, can be detrimental to the integrity of the virus particles (Gagnon, 2008; Loewe et al., 2019; Boi et al., 2020; Pereira Aguilar et al., 2020). Furthermore, porous bead-based columns are also not economical (Ghosh, 2002; Jacquemart et al., 2016) and rely on diffusion-based slower flow rates, thereby increasing the process time significantly (Ghosh, 2002).

In this study, we describe a novel, quick and cost-effective bioprocess for the purification of the oncolytic virus, VSV-GP using membrane adsorber based cation exchange chromatography. This process produces, not just a purer form of VSV-GP, but also a more concentrated drug substance. This is achieved without the need for an extensive concentration step, usually involving ultrafiltration resulting in the high concentration of oncolytic viruses, often being a pre-requisite enabling intra-tumor application of the virus. The latter is known to damage the virus particles due to shear stress (Loewe et al., 2019). Furthermore, we have characterized the purified virus using *in vitro* and *in vivo* assays to substantiate the critical quality attributes.

## Materials and methods

### Cells and viruses

BHK-21 (C13) cells used for the infectivity assay (TCID_50_) were obtained from ATCC (ATCC, 2022). 293-F suspension cells were obtained from Thermo Fisher Scientific (ThermoFisher, 2022). CT26. Cl25-IFNAR1^−/−^ cells were generated as described previously (Schreiber et al., 2019).

VSV-GP, which is VSV (Indiana strain) pseudotyped with glycoprotein from LCMV (LCMV WE HPI-GP) and has been described previously (Muik et al., 2012; Muik et al., 2014; Tober et al., 2014). A VSV-GP variant expressing firefly luciferase (VSV-GP-Luc) was also described previously (Dold et al., 2016). Viruses were amplified in 293-F cells, virus containing supernatant harvested using centrifugation at 2000 g for 5 min. Subsequently, 0.2 M NaCl is added to the suspension before filtering the material using a 0.22 µm bottle-top filter (Corning, Cat. #431098). Filtered material is then treated with SAN-HQ Nuclease (Arcticzymes, Cat. #70921-150) to reduce the host-cell DNA content. This is performed by adding 30 U/ml of SAN-HQ and incubating for 30 min at room temperature. Finally, the virus material is diluted 1:1 with 100 mM Tris-HCl, pH 7.5 to bring NaCl concentration to 0.1 M before proceeding for downstream processing.

### Cation exchange chromatography and final virus formulation

Sartobind S Nano^®^ membrane adsorbers were used for virus capture in CEX. These membranes are cellulose-based cation exchange membrane adsorber capsules with 3 ml membrane volume (MV) and a bed height of 8 mm (Cat. Reference: 96IEXS42EUC11—A, Sartorius). The nominal pore size for these membrane adsorbers is > 3 µm (Sartorius, product datasheet). For all CEX experiments, an Äkta™ Pure 150 liquid chromatography system (Cytiva) with a 280 nm UV, pH and conductivity monitoring was operated at room temperature. Prior to virus application, membranes were equilibrated with 50 mM Tris-HCl, 50 mM Arginine, pH 7.5 at a flow rate of 15 ml/min. Sample application was conducted with the same flow rate. The elution of the virus particles was carried out with a 300 mM NaCl, 50 mM Tris-HCl, 50 mM Arginine, pH 7.5 buffer at a flow rate of 3 ml/min. Pooled elution fractions from CEX were diluted 6x in order to reach the final buffer composition with 50 mM NaCl instead of 300 mM NaCl in the elution fraction. Afterwards, virus is sterile-filtered using a 0.22 µm syringe-filter (Sarstedt, Cat. #831826001). Virus aliquots were stored at −80°C before use.

### Median tissue culture infectious dose assay

To determine the quantity of infectious virus present in our samples, TCID_50_ assay was performed as described previously (Hochdorfer et al., 2022). Briefly, BHK-21 (C13) cells were plated in 96-well plates. Post-seeding (24 h), 0.5 log serial dilutions of the virus are added into the cell wells. After 3 days, cell confluence was used to determine the cytopathic effect (CPE) for each well. Cell confluencies >95% are regarded as CPE negative. Final titers (TCID_50_/ml) were obtained by the formula of Spearman Kärber (Spearman, 1908; Kärber, 1931).

### Host-cell protein ELISA assay

The quantification of host-cell protein (HCP) impurities was determined by performing the Human Embryonic Kidney 293 Host Cell Proteins ELISA kit from Cygnus Technologies (Cat. Reference: F650S) according to the manufacturer´s instructions. The amount of hydrolyzed substrate, which gives a colorimetric signal directly proportional to the HCPs concentration, was measured with a Spark 20 M Tecan Plate Reader^®^ using Nunclon^®^ Flat bottom 96-well plates (Thermo Fisher).

### Sodium dodecyl sulfate polyacrylamide gel electrophoresis and silver staining

In order to test purity and identify the viral and host-cell proteins, SDS-PAGE followed by silver staining were carried out (Chevallet et al., 2006). Precast 4%–20% gradient gels (Bio-Rad, Cat. #4561095) were chosen due to their ability of separating broad ranges of molecular weights. Virus samples were diluted with 4x Laemmli Sample Buffer (Bio-Rad, Cat. #1610747) containing freshly added 200 mM Dithiothreitol (DTT) as a reducing agent (Bio-Rad, Cat. #1610610). Afterwards, samples were heat-denatured at 95°C for 5 min. As a molecular weight ladder, Precision Plus Protein Dual Color Standard (Bio-Rad, Cat. #1610374) was used.

For revealing the gel protein bands, the commercial kit “Pierce™ Silver Stain” (Thermo Fisher Scientific, Cat. #24612) was employed. Silver staining is an ultrasensitive method which can effectively reveal protein bands that are in sub-nanogram ranges. Silver staining was performed as detailed by the manufacturer´s instructions. Gel images were acquired using VisionWorks LS software (Version 8) with ChemStudio gel imaging instrument (AnalytikJena, Jena, Germany).

### Cryo-transmission electron microscopy

Cryo-TEM imaging was performed by NanoImaging Services, Inc (San Diego, CA, United States). Each sample was prepared by applying a 3 μl drop of undiluted sample suspension to a cleaned grid (holey carbon films on 400-mesh copper grid) and immediately proceeding with vitrification in liquid ethane. Electron microscopy was performed using a Thermo Fisher Scientific Glacios Cryo-TEM operated at 200 kV and equipped with a Falcon three direct electron detector. Automated data-collection is carried out using Leginon software (Suloway et al., 2005; Cheng et al., 2021). High magnification images were acquired at nominal magnifications of 73,000 × (0.201 nm/pixel) and 28,000 × (0.519 nm/pixel) after identifying potentially suitable target areas for imaging at lower magnifications. The images were acquired at a nominal underfocus of −5.6 μm to −2.5 μm and electron doses of ∼10–25 e^−^/Å^2^.

### Analytical size exclusion chromatography

In SEC analysis, 40 µl samples were injected through a 1,290 Infinity II HPLC system (Agilent Technologies, Inc., CA, United States) with UV detection at 280 nm wavelength. Contaminant analyses were performed using TSKgel 3000 PW_XL_ SEC column (13 μm, 30 cm × 7.8 mm, TOSOH Bioscience, Tokyo, Japan) operated at room temperature with an isocratic flow at 0.5 ml/min flow rate using a mobile phase composed of phosphate buffered saline (PBS).

### *In vivo* studies

Six to eight-week-old female BALB/c (BALB/cByJ) mice, were obtained from Charles River Laboratories (Wilmington, MA, Unites States). CT26. Cl25-IFNAR1^−/−^ tumors were implanted by subcutaneous injection of 100 μl containing 1 × 10^6^ cells in the right flank (Mueller et al., 2019). Tumor size was measured three times a week with a caliper and volume was calculated using the formula: length × width^2^ × 0.5. Treatment commenced when the mean tumor volume reached a size of 80–150 mm^3^. Virus solutions with 10^9^ TCID_50_ of virus were used for intravenous (100 μl) injection. Mice were sacrificed when their tumor volume reached 1,500 mm^3^ or tumors showed signs of ulcerations (Workman et al., 2010). Animals were euthanized by overdose on gas anesthesia (isoflurane) followed by cervical dislocation or exsanguination.

### Bioluminescence imaging

For Bioluminescence imaging, a charge–coupled device camera (PhotonIMAGER RT, Biospace Lab, France) was used. Mice were injected intraperitoneally with 50 mg/kg D-Luciferin (D-Luciferin potassium salt, *in vivo* imaging solution at 15 mg/ml) in DPBS, and imaged 15 min after injection upon transient narcosis. Exposure times were adjusted to bioluminescence intensity to optimize signal levels and avoid saturation of the light sensors. For quantification of bioluminescent signal, regions of interest (ROI) were manually defined and the average intensity within the ROI was measured as photons/sec/cm2/sr. The obtained images were analyzed using M3Vision image analysis software (Biospace Lab, France).

## Results

In the current study, specific binding, and elution of oncolytic virus VSV-GP with the cationic exchange membrane adsorber, Sartobind S, was tested along with the concentration of VSV-GP from diluted crude feed material obtained after digesting nucleic acids with SAN-HQ nuclease (methods and materials). Moreover, it is important to mention that the treatment of feed with nuclease resulted in a host cell DNA concentration of only 4.16 ng/ml, which is already below any regulatory requirements (Yang, 2013). This cation exchange membrane adsorber was selected based on the virus surface properties [recombinantly modified to have LCMV GP instead of WT VSV glycoprotein (Muik et al., 2012; Kimpel et al., 2018)] having a positive net charge at physiological pH (Uniprot: P09991).

**FIGURE 1:**
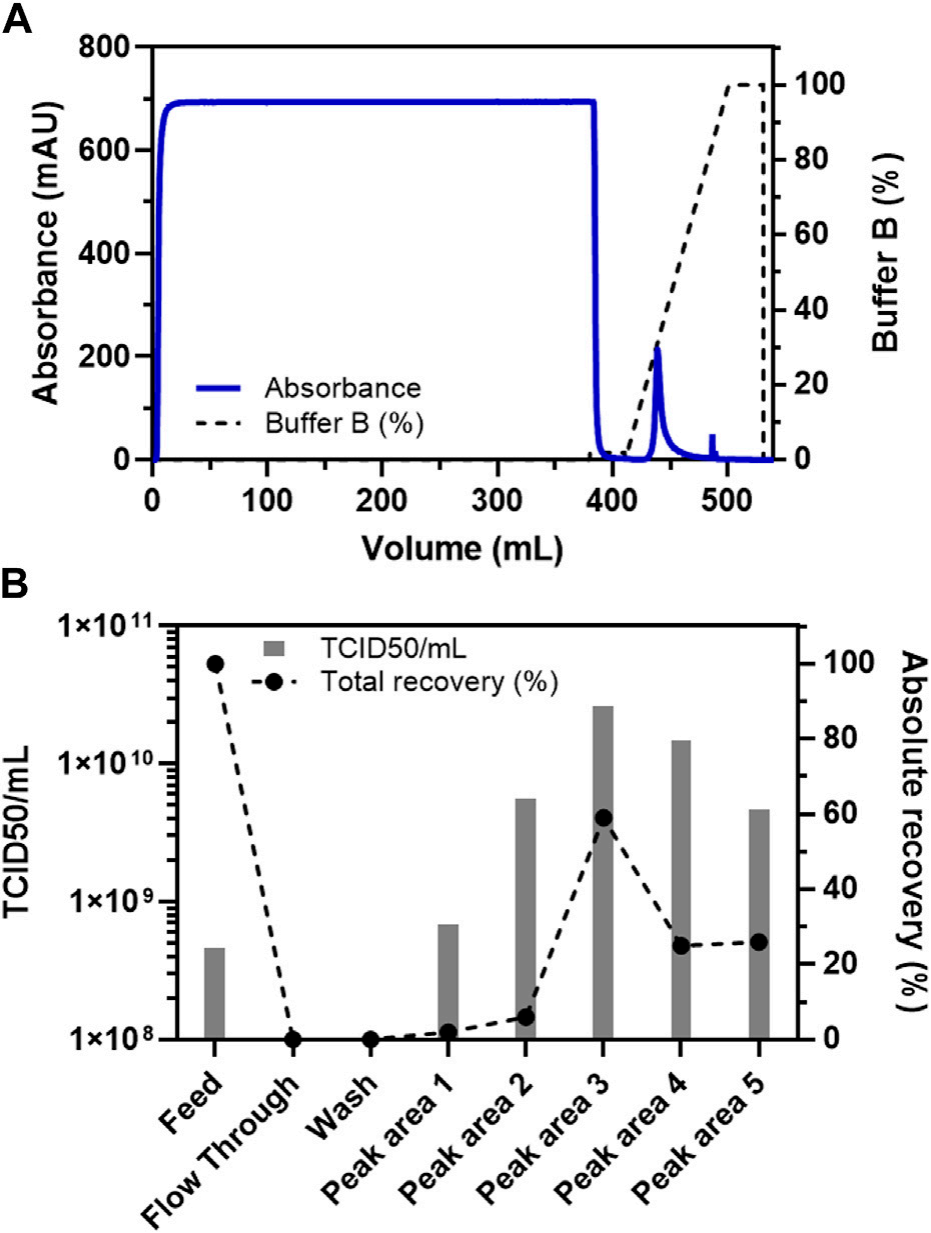
Binding and elution (using a linear gradient) of VSV-GP from the crude feed on Sartobind S membrane adsorber. **(A)** Chromatogram for the purification of VSV-GP using Sartobind S membrane adsorber column. **(B)** TCID_50_-based infectivity assay for various fractions obtained during purification of VSV-GP in **(A)**.

The current Sartobind S based chromatography method was developed to facilitate the binding of VSV-GP to the membrane adsorber column in the presence of low concentration of sodium chloride (0.1 M NaCl) thereby eliminating non-specific binding of molecules such proteins present in the crude feed (Figure 1A). A wash step, involving washing with a mild salt concentration (0.05 M NaCl), was also incorporated to further remove non-specific binders and maximize the elimination of process-related impurities, while still binding most of the quantity of the target molecule i.e., VSV-GP. Indeed, it was discovered that >95% of the virus particles bound to the column from the feed material (Figure 1). The bound material was then eluted from the column using a linear gradient of 1 M NaCl, resulting in an efficient elution (Figure 1B). The main peak observed in the elution fraction with the linear gradient was calculated to be at around 300 mM NaCl with >60% of total loaded VSV-GP particles as calculated using TCID_50_ (Figure 1).

Furthermore, we wanted to analyze the dynamic binding capacity of the column with a load of 5,800 ml of the feed material on a 3 ml Sartobind S column (Supplementary Figure S1). Despite such a high load in terms of feed volume, the number of particles detected in the flow through fractions remained relatively less, not reaching a threshold of >10% breakthrough (Supplementary Figure S1B). Observation during the linear step gradient that the main virus peak was observed at around 300 mM NaCl (Figure 1), we also incorporated a step elution gradient to pinpoint the elution profile of the VSV-GP from the cation-exchange membrane adsorber (Supplementary Figure S1). The earlier observation with the linear gradient (Figure 1A) was confirmed, showing efficient virus elution with 300 mM NaCl. Only non-significant elution occurred at higher (>300 mM) NaCl concentrations (Supplementary Figure S1).

**FIGURE 2:**
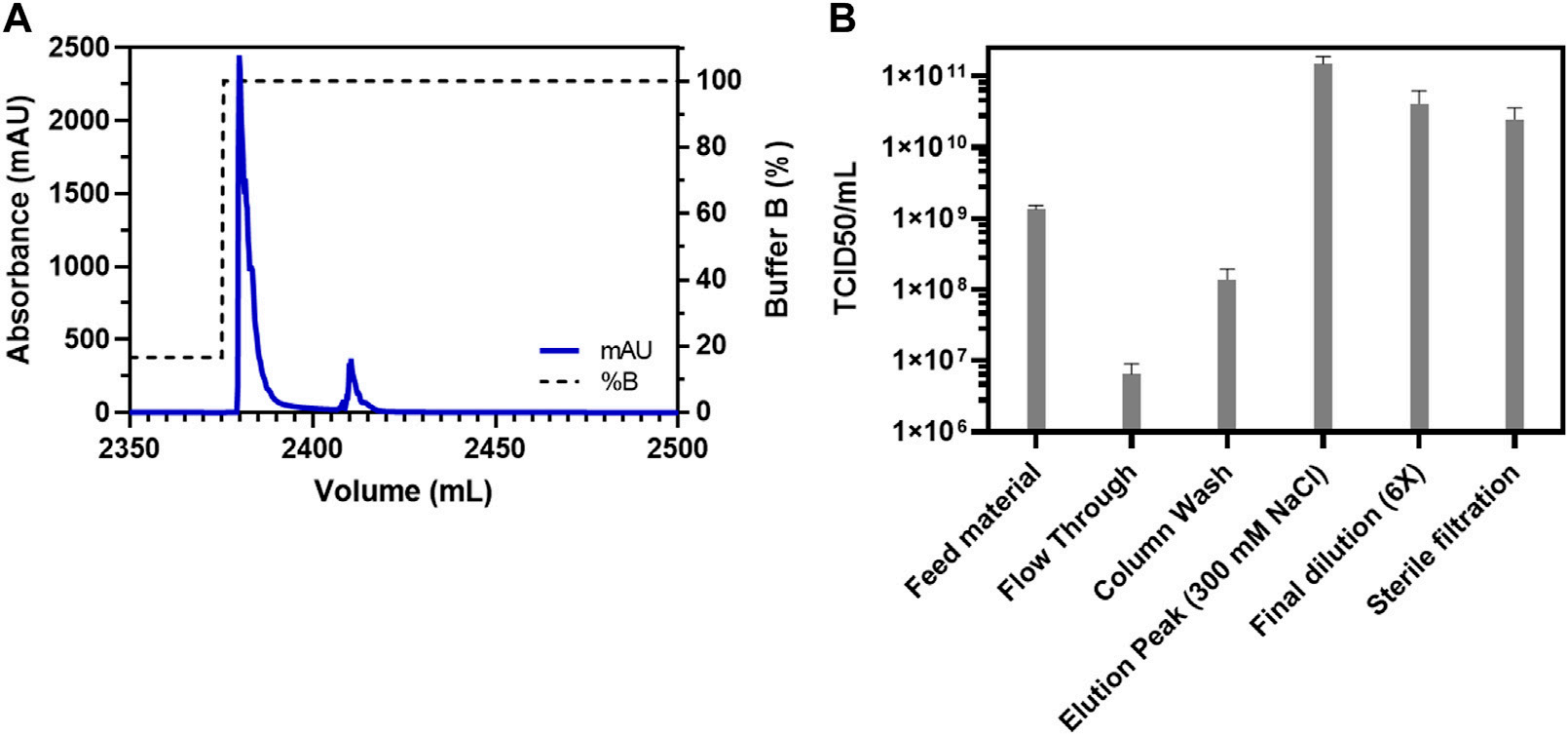
Consolidation run for the purification of VSV-GP from crude feed on Sartobind S membrane adsorber. **(A)** Chromatogram for the purification of VSV-GP using Sartobind S membrane adsorber column. The first peak contained the virus with a volume of 8 ml and **(B)** TCID_50_-based infectivity assay for various fractions obtained during purification of VSV-GP in **(A)** with final a recovery of about 30% in the elution fraction.

**FIGURE 3:**
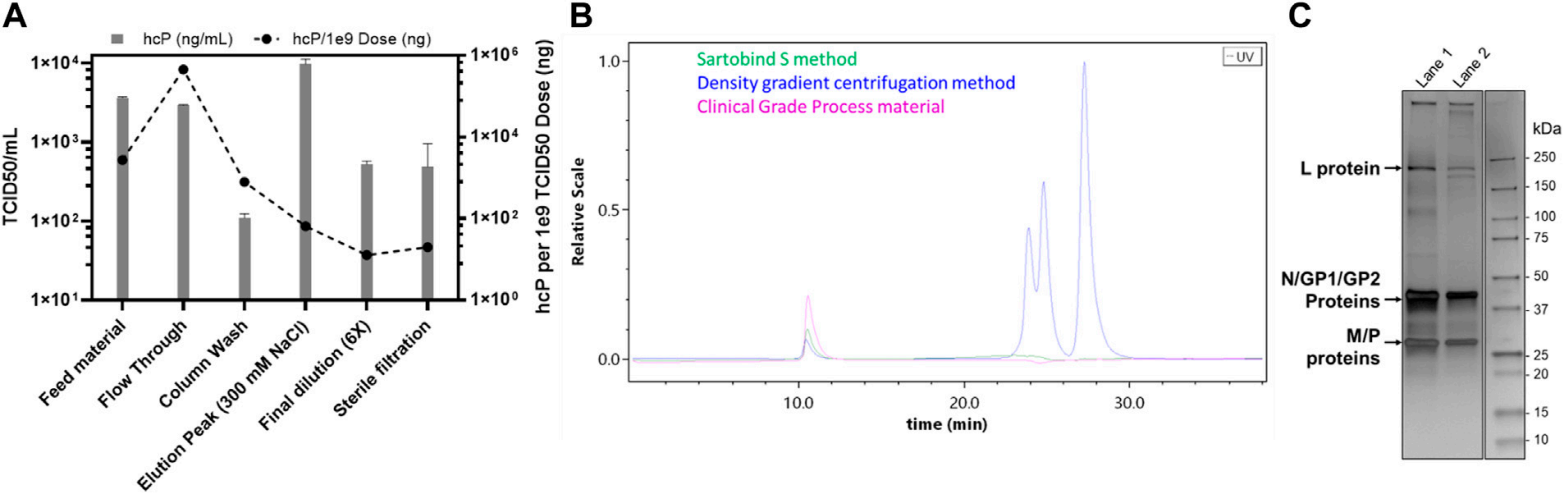
Host contaminant analysis for the purification of VSV-GP using Sartobind S membrane adsorber. **(A)** Host-cell protein ELISA for the various fractions collected during the purification of VSV-GP, **(B)** analytical SEC for the final VSV-GP preparation as compared to that of purified using a density gradient centrifugation and clinical-grade process, and **(C)** SDS-PAGE analysis, comparing the VSV-GP purified, using: density gradient centrifugation (lane 1); and Sartobind S method (lane 2). The arrows show the respective viral proteins, L: large polymerase protein; N: nucleoprotein; GP1 and GP2: processed glycoprotein complex proteins pseudotyped from LCMV; and M: matrix protein.

Apart from factors such as a higher yield, speed, and cost-effectiveness, it is also imperative to consider the purification efficiency of the process. Therefore, a consolidation run with a feed of 2,300 ml (766.66 membrane volumes) and a fixed step elution with 300 mM NaCl was carried out (Figure 2). This consolidation run was used to incorporate all previous optimizations, establish the final process, and analyze the purified virus product for its quality attributes. The consolidated process resulted in a single peak during the chromatography (Figure 2A) and a very high concentration of the virus of interest with a volume of 8 ml (Figure 2B). Generally, the requirement for oncolytic viruses is also to be administered intra-tumorally. Therefore, a higher concentration of virus in the drug product is needed, which was achieved with this step. However, the peak was further diluted 6x to have a working stock appropriate for further experiments. The purified virus material, using the Sartobind S column, was further analyzed for the removal of host cell protein impurities. The purification of crude feed material using the cation-exchange membrane adsorber resulted in a >99% removal of host cell protein contaminants (Figure 3A). Contaminant analysis using analytical SEC method (manuscript under preparation) also revealed a prominent single peak as compared to multiple peaks for the same virus purified using density gradient centrifugation (Figure 3B). SDS-PAGE analysis also revealed that the virus material was purified to a similar extent as the GMP process being used for manufacturing clinical-grade VSV-GP (Figure 3C).

**FIGURE 4:**
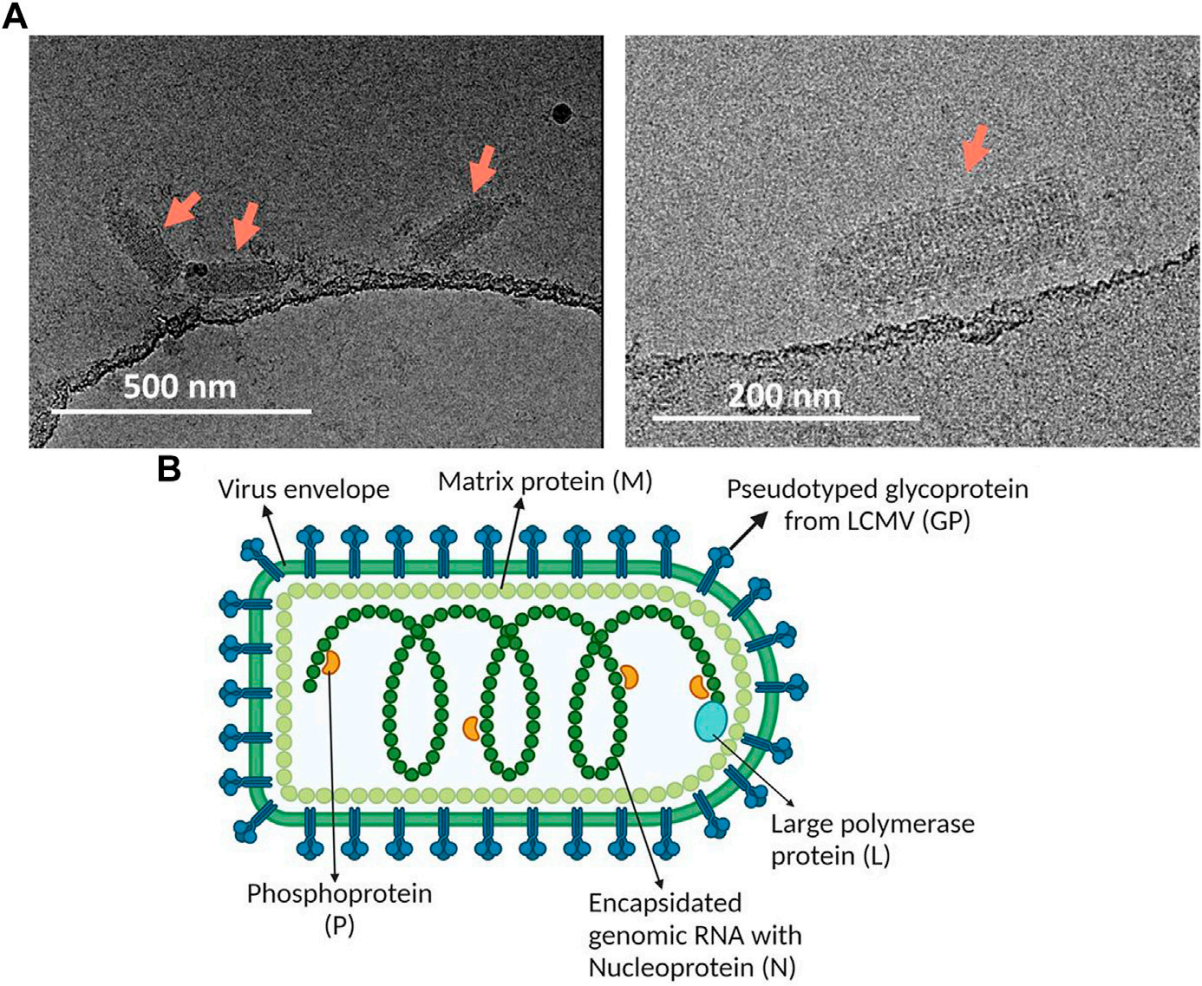
**(A)** Cryo-TEM for morphological characterization of purified VSV-GP using Sartobind S. Arrows point towards these bullet shaped VSV-GP virus particles. **(B)** Image depicting the structure of VSV-GP along with its essential structural elements. The figure was generated using BioRender.

To further substantiate the virus quality, the purified virus was analyzed using cryo-TEM (Figure 4A). Bullet shaped particles at an average size of about 190 nm × 70 nm with interior striated pattern and intact lipid bilayer membrane decorated with surface spikes were observed, consistent with features of intact VSV virus (Ge et al., 2010). VSV-GP purified using the current method correlated well with the expected bullet shaped morphology of VSV-GP virus particles, yielding native bullet shaped particles (Figure 4B).

**FIGURE 5:**
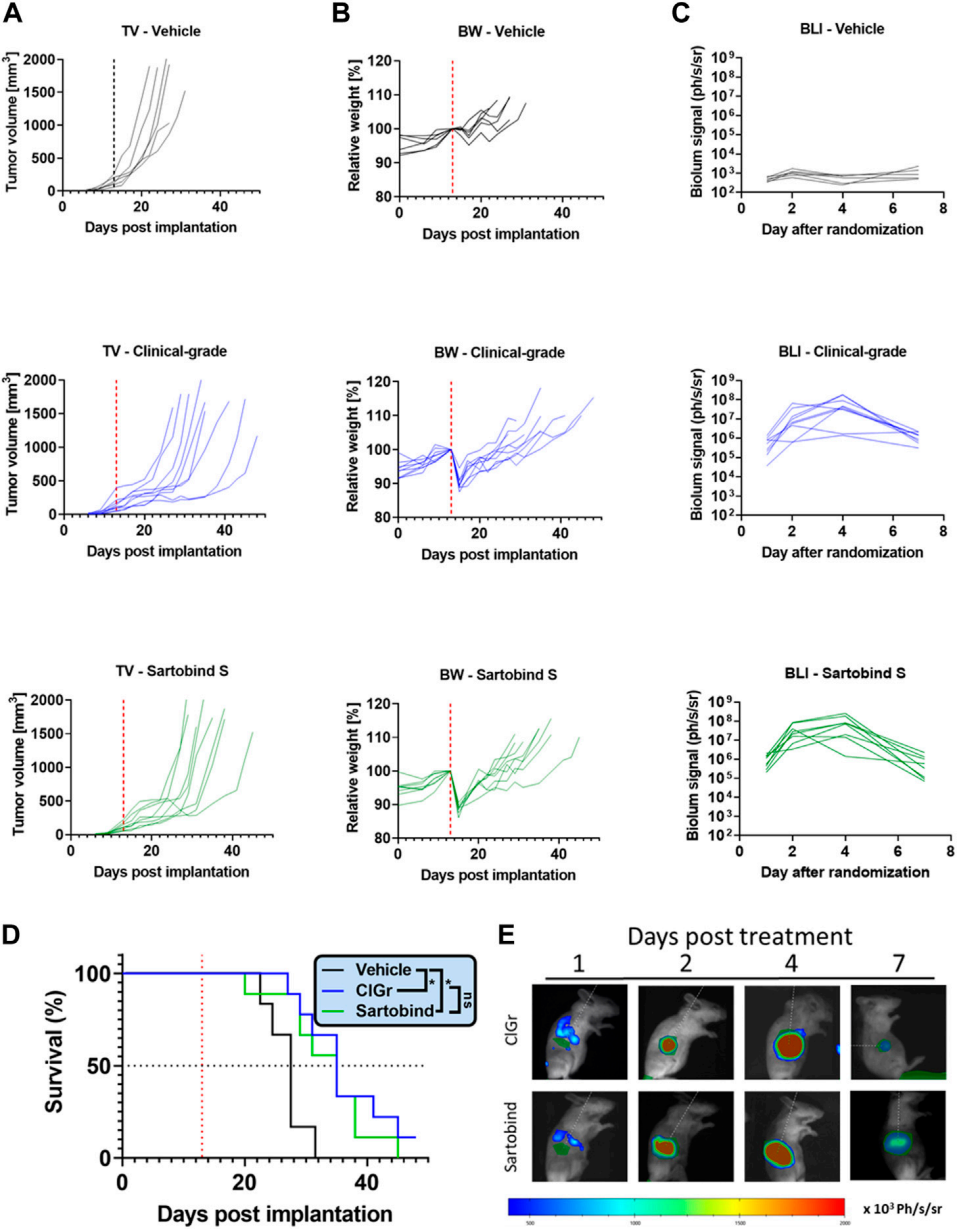
*In vivo* study compares the efficacy, tolerability, and activity of the virus purified using Sartobind S or the clinical-grade process. Tumors were implanted in BALB/c mice by subcutaneously injecting 10^6^ CT26.Cl25-IFNAR1^−/−^ cells. Established CT26.Cl25-IFNAR1^−/−^ tumors were treated intravenously with a single dose of 10^9^ TCID_50_ VSV-GP-Luc (red dotted line), produced either by the clinical-grade process (ClGr) or Sartobind S-based process (Sartobind). **(A)** Individual tumor volume (TV) graphs are shown **(B)** Individual body weight (BW) graphs are shown. **(C)** Tumors were monitored on day 1, 2, 4, and 7 post treatment using the *in vivo* bioluminescence imaging (BLI) system IVIS. Quantification of the average radiance in the individual tumor areas are shown. **(D)** Kaplan–Meier survival curves are shown. Virus treated animals showed a significant survival benefit. Log-rank (Mantel-Cox) test was performed (*p < 0.05) **(E)** Representative BLI pictures of treated mice are shown. A region of interest (ROI) was drawn on bioluminescent images over the subcutaneous tumors. Luminescence is displayed as photons/second/steradian (p/s/sr).

Finally, we wanted to ascertain that the therapeutic virus was effective in animal models *in vivo*. Therefore, we tested and compared it with the virus material purified using a comprehensive downstream process (clinical-grade process) currently used for manufacturing at large scale for clinical trials. To address whether the different downstream processes have an impact on the efficacy, tolerability, or activity of the virus, we compared relevant read-outs in BALB/c mice bearing CT26. cl25-IFNAR1^−/−^ tumors (Figure 5). A single systemic VSV-GP-luc injection of 10^9^ TCID_50_ induced a delay in tumor growth with no difference between our new and clinical-grade process (Figure 5A). This resulted in an identical mean survival benefit of 8 days compared to vehicle-treated animals (Figure 5D). Both test substances were also well tolerated with only a transient weight drop being reported from which all animals recovered within in few days (Figure 5B). After treatment, virus activity was monitored over time *via* luciferase signal. The respective treatments resulted in comparable tumor-selective bioluminescence signals. These signals increased over several days, peaking at day 4, indicating similar active viral replication (Figures 5C,E).

## Discussion

Cation exchange chromatography is a purification platform used frequently for purification of not only proteins and antibodies (Brämer et al., 2019; Boi et al., 2020), but can also be efficiently utilized for the purification of complex viruses, as demonstrated in this study and previously by other authors (Eckhardt et al., 2022; Mueller et al., 2022). For example, Eckhardt et al. (Eckhardt et al., 2022) have used resin based cation exchange chromatography for the purification of measles virus. However, apart from dis-advantages such as lower flow rates limited by diffusion, resin based chromatography media also have drawbacks in terms of disrupting the integrity and infectivity of virus particles due to increased shear stress, especially if process times are rather long (Gagnon, 2008). Moreover, adopting a protocol from one virus to another is extremely challenging and cannot be easily adopted. As it depends on multiple factors such as surface glycoproteins, cell lines used to generate the virus particles, enveloped vs. non-enveloped virus particles, size and shape of the virus particles, etc. (Nestola et al., 2015; Zhou et al., 2020; Wright et al., 2022). Mueller et al. (2022) in their patent application have described a process for the purification of VSV-GP however the current study demonstrates a single step (vs multi step) process using membrane adsorber (vs monolithic) chromatography columns.

There are other chromatographic methods for the purification of certain viruses, such as affinity chromatography which can be highly specific for the target particles with very good removal of contaminants without additional polishing steps (Zhao et al., 2019). However, the disadvantages still outweigh the advantages especially when it comes to the purification of new modalities such as large virus particles, i.e., VSV-GP. The drawbacks include slow and long process due to the time required to develop the affinity molecules or antibodies, scale up challenges, non-optimal reproducibility due to lack of streamlined chromatographic matrix-affinity ligand production, lack of elution due to strong multivalent interaction, etc. Emergence of pseudo-affinity ligands and chromatographic media in the market might tackle some of these challenges but that is something still to be validated (Opitz et al., 2009). Another type of chromatographic technique has emerged for the purification of viruses called steric exclusion chromatography (Lothert et al., 2020). However, this technique also lacks due to the absence of a streamlined raw material supply, scale up and industrial applications, as well as requirement of high viscosity materials during chromatography such as polyethylene glycol (PEG). Ion exchange chromatography coupled with convective flow chromatographic matrix (i.e., membrane adsorber) overcomes these challenges, as it is a well-established technique not just for lab scale but also for industrial scales.

In this study we utilized the capabilities of cation exchange chromatography coupled with convective flow media, i.e., membrane adsorber. This is a very important single use technology along with large pore sizes/channels (Hagemann et al., 2022), allowing an efficient capture/binding of the virus of interest, as well as their efficient purification. The yields obtained for the purified virus (VSV-GP) were also impressive using the method in this study, as compared to other processes (Mueller et al., 2022), while retaining critical quality attributes. Additionally, there was a good reproducibility of the method even with different working volumes of feed when applied to the column along with any observed absence of membrane clogging or fouling (Figures 1, 2). Therefore, this study demonstrates that a simple downstream process can be developed and implemented for the purification and concentration of ATMPs, such as oncolytic viruses. Complex multi-step processes can easily result in reduced infectious virus yields and quality. However, both the yield and quality are extremely important for applications such as oncolytic viruses. Therefore, the current process provides the benefits of a lean downstream process, combined with the higher product quality and yield.

## Data Availability

The original contributions presented in the study are included in the article/Supplementary Material, further inquiries can be directed to the corresponding author.
