# Supplementary material for: Single-step rapid chromatographic purification and characterization of clinical stage oncolytic VSV-GP
Source: Front Bioeng Biotechnol. 2022 Oct 28;10:992069. doi: 10.3389/fbioe.2022.992069 (PMC9649487; doi:10.3389/fbioe.2022.992069)
Supplement: Supplementary file 1 [file Image1.pdf]

Supporting Information

Single-step rapid chromatographic purification process and characterization of clinical stage oncolytic VSV-GP

Saurabh Gautam[1,2]*, Dongyue Xin[3], Alan Pardo Garcia[1,2], Bart Spiesschaert[1,2]

[1]Boehringer Ingelheim International GmbH, 55216 Ingelheim, Germany

[2]ViraTherapeutics GmbH, 6063 Rum, Austria

[3]Boehringer Ingelheim Pharmaceutical, Inc., Ridgefield, CT06877, USA

*Corresponding author

Saurabh Gautam

saurabh.gautam@boehringer-ingelheim.com; gautamsaurabh12@gmail.com

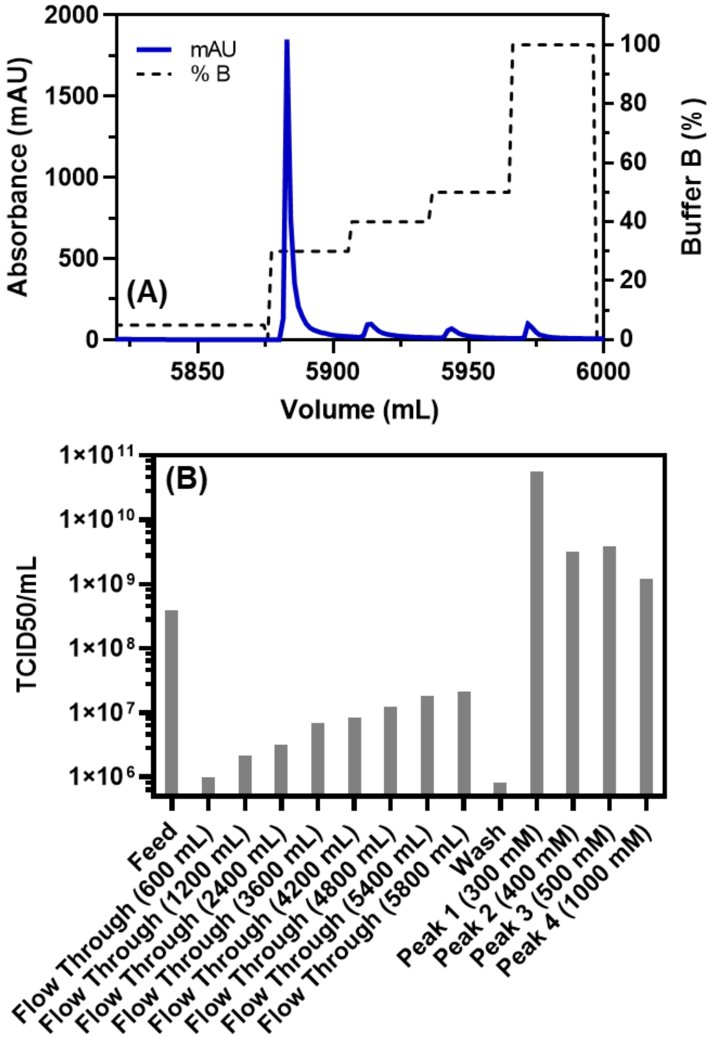

Fig. S1. Loading and elution of 5800 mL of crude feed containing VSV-GP on Sartobind S membrane adsrober column. (A) Chromatogram for the purification of VSV-GP with Sartobind S membrane adsorber column using a step elution gradient. (B) TCID50 based infective assay for various fractions obatined during purification of VSV-GP in (A).
